# Partial hydatidiform mole and coexisting fetus after frozen embryo transplantation: a case report

**DOI:** 10.5935/1518-0557.20230069

**Published:** 2024

**Authors:** Jinran Li, Xiaoli Sun

**Affiliations:** 1 Affiliated Hospital and Medical School of Nantong University, Jiangsu, China; 2 Sun XL is the corresponding author, Reproductive Medicine Center of Affiliated Hospital of Nantong University, People’s Republic of China

**Keywords:** FET, hydatidiform mole and coexisting fetus, multiple embryo transfers

## Abstract

Hydatidiform mole and coexisting fetus is a very rare condition of which etiology is still inconclusive. It may occur after assisted reproduction, often leading to the death of normal embryos and other serious complications. We report a case of partial hydatidiform mole and coexisting fetus after frozen embryo transplantation. More than two months after the patient underwent transplantation with two blastocysts (scored 4AB and 4BC), B-ultrasound showed a single live fetus with a large dense dotted strong echo area. The patient was treated with chemotherapy after the termination of pregnancy due to persistently increased human chorionic gonadotropin levels. Many studies have described trophoblast quality as a strong predictor of pregnancy. In the case in question, in addition to partial hydatidiform mole caused by multiple sperm entering the egg, we also speculate that the condition may be related to the poor quality of the trophoblastic ectoderm of the transferred embryo. In the process of assisted reproduction, the transfer of embryos with poor trophoblastic ectoderm in multiple embryo transfers may adversely affect pregnancy outcomes.

## INTRODUCTION

Hydatidiform mole is a common gestational trophoblastic disease, characterized by abnormal embryo development, chorionic edema and degeneration, and trophoblastic hyperplasia. The disease is sporadic in Western countries, and incidence in developing and underdeveloped countries is about 2 to 10 times higher (Deveault *et al*., 2009; Akoury *et al*., 2015). In China, the incidence of hydatidiform mole is about 1‰~8.83‰, with Zhejiang Province ranking first (Qian *et al*., 2011).

Hydatidiform mole and coexisting fetus is a very rare disease. The fetus may survive despite the occurrence of severe maternal-fetal complications such as preeclampsia, thromboembolic disease, pregnancy vomiting, hemorrhage, although intrauterine fetal death may also occur (Ferraz *et al*., 2013). Hydatidiform mole during pregnancy including partial hydatidiform mole (PHM) and complete hydatidiform mole (CHM) with a normal fetus have been described. At the cytogenetic level, more than 90% of PHMs are triploids formed by double spermatozoa and one ovum, i.e., one chromosome from the mother and two from the father (Kalogiannidis *et al*., 2018). In CHMs, 80% to 90% of spermatozoa are haploid, i.e., fertilized with a chromosomally inactivated ovum or an empty ovum, or a chromosomally inactivated ovum or an empty ovum fertilized by a diploid sperm from a failed second meiosis (Vassilakos *et al*., 1977).

Here, we share a case of partial hydatidiform mole and coexisting fetus (PHMCF) after frozen embryo transplantation.

## CASE REPORT

The patient is a 32-year-old female, received the second in vitro fertilization (IVF) treatment due to a “history of bilateral salpingectomy”. The couple had received frozen embryo transfers twice in the external hospital five years before, but did not become pregnant.

The pre-IVF examination did not show any significant abnormalities. The antagonist regimen was used to promote ovulation and 13 oocytes were obtained, 8 of which were of fair maturity. After fertilization, fresh embryo transplantation was not performed due to posterior fornix tenderness. Two D3 embryos were frozen and the remaining embryos were bred, and a total of 7 blastocysts were formed.

**Figure 1:**
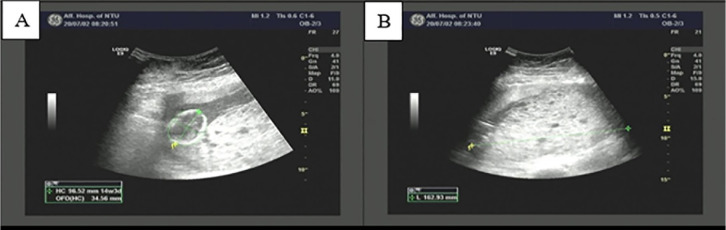
B-ultrasonography at 20 weeks of gestation showing intrauterine singleton and hydatidiform mole tissue. A. Intrauterine single live fetus; B. Large dense dot shaped strong echo area in uterine cavity (167 × 43 × 159mm), indicating partial hydatidiform mole.

In the first cycle, two Day3 secondary-level embryos were transferred but ended in failure. In the second cycle, two blastocysts were transplanted, and the scores were 4AB and 4BC, respectively. Serum human chorionic gonadotropin (HCG) was 373.6 U/L 10 days after embryo transplantation. More than one month after transplantation, B-ultrasound showed a 9×6mm gestational sac-like echo beside the intrauterine singleton pregnancy, without a yolk sac or germ. On the 87^th^ day of pregnancy, B-ultrasound showed a single live fetus, anterior wall placenta, normal amniotic fluid volume, and a large dense dot shaped strong echo area in the uterus, indicating partial hydatidiform mole and coexisting fetus (Figure 1). The patient had no conscious symptoms, and the pregnancy was terminated in the external hospital. Histological examination confirmed the previous diagnosis. Chemotherapy was given because HCG remained higher than normal after the procedure, and no gene mutation was detected.

## DISCUSSION

The etiology of hydatidiform mole is still inconclusive, and many scholars believe that it is a multi-step process involving multiple genes, including the activation of proto-oncogenes and oncogenes, as well as the alteration of telomerase activity and abnormal expression of matrix metalloproteinases (Liu & Zhang, 2006). Scholars analyzing previous cases found that, in addition to genetic factors, there was also a correlation between the incidence of hydatidiform mole, patient age, and number of pregnancies. Women aged 25-29 have the lowest incidence of hydatidiform mole (Bagshawe *et al*., 1986), while pregnant women older than 35 years have a 2-fold increase in incidence (Parazzini *et al*., 1986). The prevalence of the condition in first pregnancies was also much lower than in subsequent pregnancies (Acaia *et al*., 1988; Deng, 2011).

Triploid fetuses in PHM usually have multiple malformations and severe asymmetric fetal growth restriction before abortion or fetal or neonatal death. PHM may also develop into a trophoblastic tumor, and most of them are triploid karyotype. Therefore, once PHMCF is diagnosed, pregnancy should be terminated immediately; If the family strongly opposes the termination of pregnancy, the karyotype must be determined. If the karyotype is diploid, the pregnancy can continue, but serum HCG levels must be closely monitored during pregnancy and close attention paid to whether the lesion develops into a gestational trophoblastic tumor; If it is triploid or polyploid, the pregnancy must be terminated immediately.

Complete hydatidiform mole and coexisting fetus (CHMCF) is extremely dangerous for both mother and fetus, with an incidence of 1/20,000 to 1/100,000 (Steller *et al*., 1994). Only a few cases have been reported globally, approximately a third occurring after assisted reproduction. It has been reported that the increased incidence of medically induced multiple pregnancies may lead to a concomitant increase in the incidence of CHMCF (Matsui *et al*., 2000). If CHMCF continues, the chance of developing a gestational trophoblastic tumor is as high as 55% (Steller *et al*., 1994). The rate of malignant transformation is much higher than that of PHMCF. In the past, termination of pregnancy was the only treatment option, but in recent years there have been reports of successful pregnancies after CHMCF (Alpay *et al*., 2021).

The two frozen embryos transferred in this case were obtained by standard IVF, and the staff did not observe the pronucleus and polar bodies of the embryos on the first day after fertilization. Therefore, it cannot be ruled out that triploid or polyploid karyotypes were caused by multiple sperm entering the egg, which eventually developed into PHMCF. While intracytoplasmic injection of monosperm (ICSI) ensures the entry of monosperm into the egg, the induction of superovulation and other factors leading to empty follicles without nuclei increase the possibility of CHM (Nobuhara *et al*., 2018). In addition, ICSI avoids the production of triploids and polyploids, which can theoretically avoid the risk of most PHMs. However, a few PHMs are caused by diploid sperm fertilization or the replication of paternal haploid genome after fertilization. We also noticed a case of PHM after ICSI (Wood *et al*., 2002). The authors basically ruled out the above possible mechanisms in their analysis, and posited that the differentiation of the inner cell mass occurred earlier than the formation of the ectoderm, which led to the abnormal formation of trophoblasts from the inner cell mass and the production of partial hydatidiform mole vesicles with loose primitive mesoderm. We also tried to explore the possible mechanism of PHMCF in this case from the selection of inner cell mass (ICM) and trophoblastic ectoderm (TE) for quality.

In assisted reproduction, the selection of embryos depends on the quality of ICM and TE, which are coordinated to finely regulate blastocyst development and embryo implantation (Zhao *et al*., 2017). A retrospective study (Xia *et al*., 2019) reported that the miscarriage rate of embryos without high quality ICM and TE was lower in CB-grade embryos than in BC-grade embryos. At the same time, the clinical pregnancy and live birth rates increased significantly and the miscarriage rate decreased significantly with increasing TE scores relative to the morphology and grade of ICM. Logistic regression analysis showed that the quality of trophoblast cells was a good predictor of the rate of continued pregnancy in patients (Wang *et al*., 2015). In other words, the TE grade is a better predictor of the outcome of pregnancy than the ICM grade. Biopsy performed in trophoblast cells before frozen embryo transplantation (He *et al*., 2022) found that genetic testing before transplantation did not increase the risk of adverse pregnancy outcomes.

However, this was a single-center, retrospective study with a small sample size, with conclusions that need to be further confirmed by a multicenter, large sample size study. In our case, the patient received two embryos, grade 4AB, and 4BC, respectively. The quality of one embryo was not high, with a TE grade of C, according to the embryo grading. Hydatidiform mole has been described as a disease of placental trophoblast origin that may develop from trophoblast cells through multi-gene and multi-step alterations, with incidence also correlated with patient age and pregnancy history. We cannot ascertain that the embryo that developed into a partial hydatidiform mole in our case was an embryo with a low grade of trophoblastic ectoderm. We may speculate, however, that it was the trophoblastic ectoderm with reluctant quality of this embryo that changed accordingly during the subsequent pregnancy and eventually developed into a situation where a partial hydatidiform mole coexisted with a normal fetus. This may also suggest that the use of multiple embryo transplantations simply to improve pregnancy success rates is not entirely the right direction in assisted reproductive technology embryo transplantation, and reluctant transplantation of poorly graded embryos may affect the developmental process of high-quality embryos, resulting in the waste of high-quality embryos and possibly malignancy.
